# Supplementary material for: Motivation biases behavior but not perception
Source: Commun Psychol. 2026 Apr 26;4:72. doi: 10.1038/s44271-026-00461-4 (PMC13110355; doi:10.1038/s44271-026-00461-4)
Supplement: Supplementary file 2 — Supplementary Material [file 44271_2026_461_MOESM2_ESM.pdf]

Supplementary Information

Supplementary Table 1.

Deviation from preregistration in Experiment 1.

aspect	preregistration	deviation
eye movement outlier classification	For the eye movement analysis, we will not consider trials with missing data during the saccade (e.g., due to blinks)	To compute mean endpoints for each participant, we not only removed trials with blinks during the saccade, but additionally removed trials with unrealistic values regarding saccade duration or peak-velocity. Specifically, trials in which saccade duration exceeded 100 ms or saccade peak-velocity exceeded 1000 deg/s. These extreme values are most likely artefacts that arise due to (partial) data loss.
Exploratory analysis: saccadic adaptation	Additionally, we will explore whether we find evidence of saccadic adaptation, i.e. a shift of eye movement endpoints over the time course of the experiment (e.g. towards the high value location) or systematic trial-to-trial changes, e.g. towards the disc location that was task-relevant in the previous trial.	We observed no evidence for adaptation of saccades. We do not report this exploratory analysis.
Frequentist vs Bayesian statistics	To test hypotheses H1-4, we will use t-tests.<br>To test hypotheses H5-10, we will use linear regressions.	We complemented our analyses with Bayesian statistics (Bayes Factors) using the equivalent Bayesian tests, either Bayesian t-tests or Bayesian regressions.
Attentional imbalance		Given that attentional thresholds were not normally distributed (Fig. 3C), we computed an attentional imbalance score (see Methods): First, we subtracted the low value from the high value threshold. Then, we divided by the sum of both thresholds.<br>This score was used (i) to test whether there is a difference in attentional thresholds, (ii) to test the relationship with the RAI, as well as (iii) within the mediation models.

Supplementary Table 2.

Comparison between parametric and non-parametric tests for illusions in Experiment 4.

**i**llusion	**p**arametric test (t-test)	**N**on-parametric test (Wilcoxon-signed rank)
aggregated (all illusions)	$t(23) = 4.12$, $p < 0.001$, d = 0.84, $BF_{10} = 76.45$	$W = 207.5$, $p < 0.001$, $r = 0.98$
Seal-donkey	$t(23) = 1.68$, $p = 0.107$, $d = 0.34$, $BF_{10} = 0.72$	$W = 52.0$, $p = 0.092$, $r = 0.58$
Duck-rabbit	$t(23) = 2.22$, $p = 0.036$, $d = 0.45$, $BF_{10} = 1.68$	$W = 33.0$, $p = 0.036$, $r = 0.83$
Young-woman-old-woman	$t(23) = 3.73$, $p = 0.001$, $d = 0.76$, $BF_{10} = 32.27$	$W = 91.0$, $p = 0.001$, $r = 1.00$
B-13	$t(23) = 4.16$, $p < 0.001$, $d = 0.85$, $BF_{10} = 83.41$	$W = 131.5$, $p < 0.001$, $r = 0.93$

Note. For Wilcoxon-signed rank tests, the effect size is given by the rank-biserial correlation, r.

Supplementary Methods. Translation of the C-RAI questionnaire into German. For this study, we translated the scale into German (i.e., the participants' native language) using of a forward-back procedure[1]. Altogether, the translation process comprised five steps: First, the scale was translated into German using an AI tool (ChatGPT version 3.5). The resulting translation was validated by one of the authors who is a native English speaker and fluent in German (HR). In a second step two of the authors (CW, ML), who are native German speakers and fluent in English, independently revised the translation. Third, all authors agreed on a common translation for the three items that did not match after step 2. In a fourth step, a person naïve to the questionnaire and the purpose of this study translated the German items back into English. Deviations from the English original were found in two items. In a fifth step, the three authors therefore revised one of the German items, and considered the other item to be conceptually equivalent to the original despite the lexical deviation. The translated subscales demonstrated good reliability in all experiments (Experiment 1: $\alpha_{controlled} = 0.79$, $\alpha_{autonomous} = 0.81$; Experiment 2: $\alpha_{controlled} = 0.81$, $\alpha_{autonomous} = 0.91$; Experiments 3–4: $\alpha_{controlled} = 0.84$, $\alpha_{autonomous} = 0.89$). The individual steps of the translation process can be retraced from a document on the project's OSFs webpage (https://doi.org/10.17605/OSF.IO/2S5V6). In all experiments, participants were asked to rate their agreement with statements in relation to their motivation for achieving the goal of the task.

Supplementary Note 1. Mediation models. Sample size for Experiment 1 was determined a priori using Monte Carlo power simulations (N = 1,000)[2]. Assuming moderate standardized path coefficients ($\beta = .35$), a sample of 60 participants provided >80% power ($\alpha = .05$) to distinguish between a serial and a parallel mediation model. These analyses informed the original study design and are reported here for completeness. In Experiment 1, we found no evidence for a direct relationship between perceptual sensitivity and motivational quality, $F(1, 58) = 0.083$, $p = 0.774$, $BF_M = 0.271$. We therefore tested two path models to assess how perceptual sensitivity is explained by motivational quality (i.e., C-RAI score), attentional imbalance, and eye movements. Specifically, we tested a parallel model – wherein attentional imbalance and eye movements separately mediate associations between motivational quality and perceptual sensitivity – against a serial mediation model – wherein motivational quality predicts eye movement behavior, which predicts attention, and in turn perceptual sensitivity. In both models, the dependent variable was the difference in d' between the high and low value location. We assessed the goodness of each model fit using the RMSEA (root mean squared error of approximation), the SRMR (standardized root mean square residual), the CFI (comparative fit index), χ^2. Models were compared using a likelihood ratio test.

The parallel model (BIC = 330.82, RMSEA < 0.001, SRMR = 0.043, CFI = 1, $\chi^2(1, N = 60) = 0.79$, $p = 0.374$) outperformed the serial model (BIC = 361.39, RMSEA = 0.528, SRMR = 0.208, CFI = 0, $\chi^2(1, N = 60) = 35.45$, $p < 0.001$), $\Delta\chi^2 = 34.66$, $p < 0.001$, with the only meaningful relationship being the relationship between eye movements and perceptual sensitivity, $\beta = 0.556$, $p < 0.001$ (Supplementary Figure 1).

Supplementary Note 2. The relationship between eye movements and d'. A relationship on the group-level does not necessarily imply that the same relationship also exists on the individual level (ergodicity problem). Thus, to further ensure that eye movement behavior affected d' values, we additionally assessed the relationship between endpoints and d' within individuals. Therefore, we combined individual data from the high and low value condition and computed the distance from the sampled location. For example, a vertical endpoint of 0 would be 3 deg away from both, the high value and the low value location, whereas an endpoint of -1 deg would be 4 deg away from the high value location and 2 deg away from the low value location. In a second step, the individual data were split into 5 bins (<1.5 deg, 1.5–2.5 deg, 2.5–3.5 deg, 3.5–4.5 deg, >4.5 deg) and d' was computed for each bin. We regressed d' values on the endpoint distance, treating bins with less than 5 data points for either the hit rate or false alarm rate as missing values. The average slope was M = -0.25 (SD = 0.26), $t(59) = 7.41$, $p < 0.001$, $d = 0.96$, *95% CI* [0.65, 1.26], $BF_{10} = 3.89\times10^7$, indicating that d' decreased by 0.25 when a saccade was 1 deg further away from the sampled location.

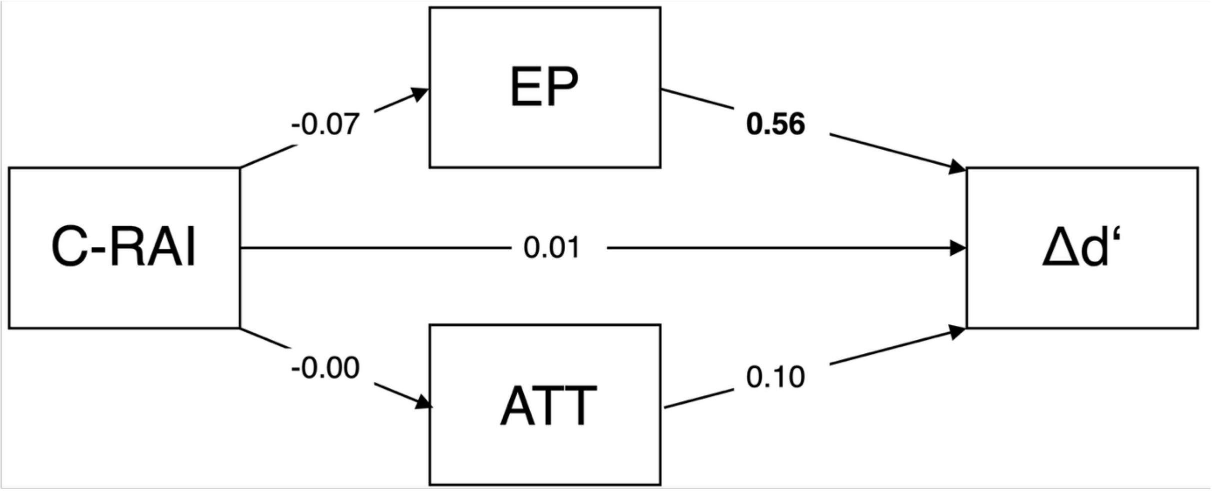

Supplementary Figure 1. Parallel mediation model. C-RAI: Relative autonomy index, EP: vertical endpoint, ATT: Attentional imbalance. Bold values indicate a meaningful relationship.

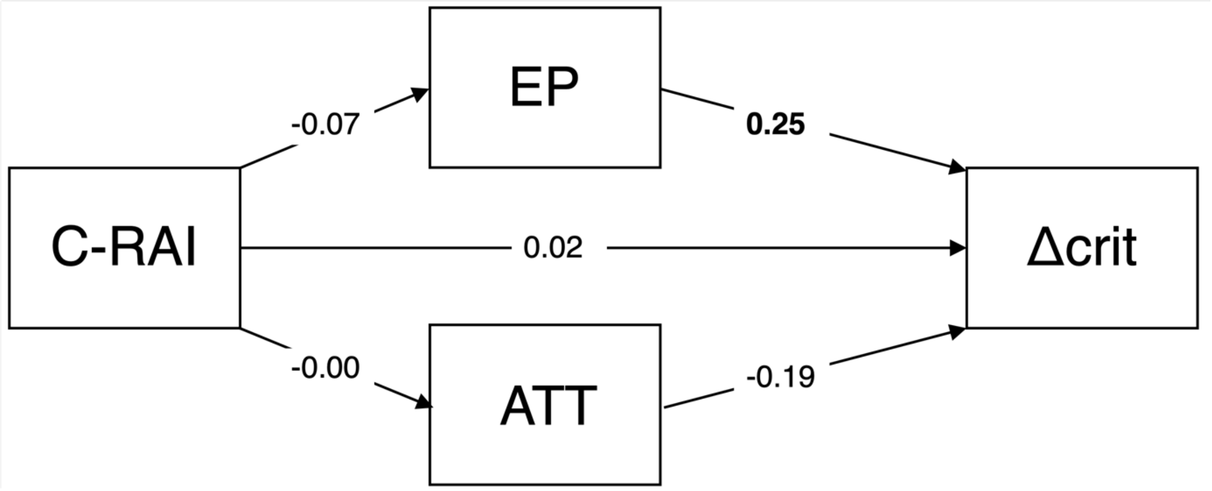

Supplementary Figure S2. Mediation model with criterion differences as dependent variable. C-RAI: Relative autonomy index, EP: vertical endpoint, ATT: Attentional imbalance, Δcrit: criterion difference (high-value minus low-value). Bold values indicate a meaningful relationship.

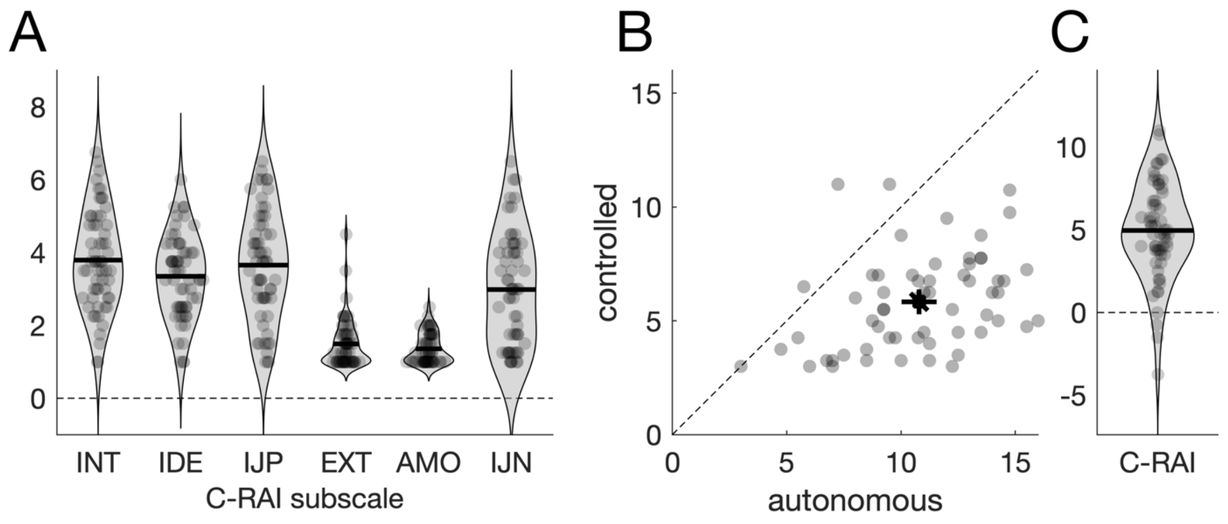

Supplementary Figure 3. C-RAI subscales. (**A) V**iolin plot for the six C-RAI subscales, INT: intrinsic, IDE: identified, IJP: positive introjection, EXT: external, AMO: amotivated, IJN: negative introjection, with the sum of the former three yielding the value for the combined scale autonomous, and the latter three yielding the controlled scale. (**B**) Scatter plot for the controlled versus autonomous scale (n = 60). Gray dots denote individuals, the black data point is the overall mean with 95% confidence intervals. The diagonal error bar denotes the difference between autonomous and controlled motivation and must be compared to the identity line. (C) Violin plot of the resulting C-RAI score (autonomous minus controlled).

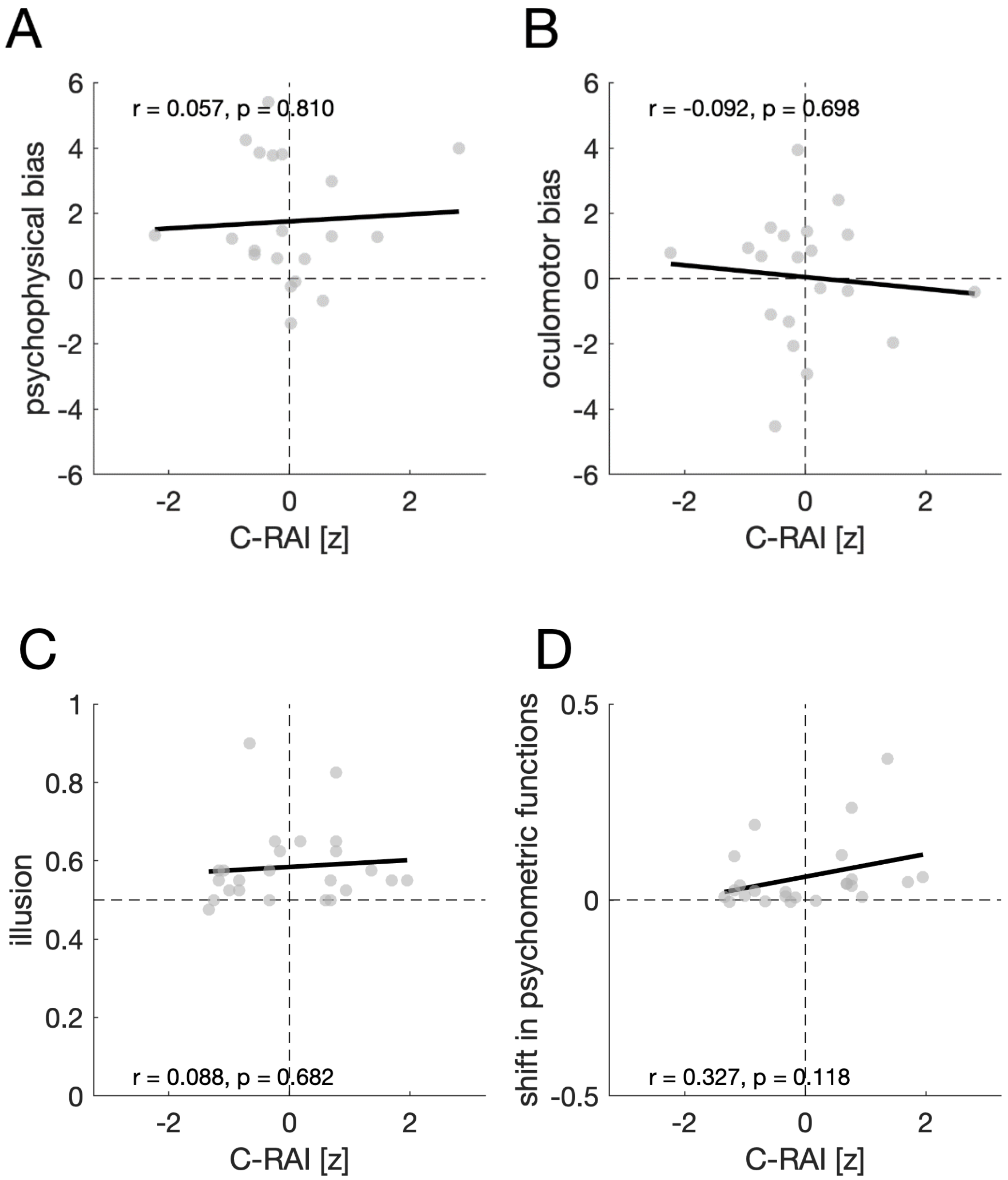

Supplementary Figure 4. No effect of goal-directed motivational quality in Experiments 2 & 4. Relative autonomy indices (C-RAI) were neither related to (**A**) the psychophysical bias (n = 20), nor to (**B**) the oculomotor bias (n = 20), nor to (**C**) the bias observed in visual illusions (n = 24) or (**D**) the bias observed in composite images (n = 24). Gray data points denote the data of individuals, the solid black line the linear regression through the data.

Supplementary References

1. Toma, G., Guetterman, T. C., Yaqub, T., Talaat, N. & Fetters, M. D. A systematic approach for accurate translation of instruments: Experience with translating the Connor–Davidson Resilience Scale into Arabic. *Methodol. Innov.* **10**, 2059799117741406 (2017).
2. Schoemann, A. M., Boulton, A. J. & Short, S. D. Determining Power and Sample Size for Simple and Complex Mediation Models. *Soc. Psychol. Personal. Sci.* **8**, 379–386 (2017).
